# Role of Multidetector Computed Tomography for Evaluation of Living Kidney Donors

**DOI:** 10.5812/numonthly.10875

**Published:** 2013-08-12

**Authors:** Baratali Asghari, Mansour Babaei, Bijan Pakroshan, Alireza Vaziriniya, Abdolreza Babamahmoodi

**Affiliations:** 1 Health Management Research Center, Baqiyatallah University of Medical Sciences, Tehran, IR Iran; 2 Urology Department, Besat Hospital, Tehran, IR Iran

**Keywords:** Kidney Transplantation, Multidetector Computed Tomography, Tissue Donors

## Abstract

**Background:**

Kidney transplantation from living donors has been increased recently. Preoperative evaluation of living donor is important to select the appropriate kidney for transplantation and to decrease donor surgical complications.

**Objectives:**

The aim of this study was to compare the accuracy of the use of multidetector computed tomography (MDCT) to evaluate vascular anatomy in living kidney donors with traditional angiography.

**Patients and Methods:**

A total number of 60 living kidney donors who underwent open surgical approach for transplantation were selected: Kidney anatomy of donors evaluated by CT angiography (group 1) or traditional angiographic examination (group 2). Renal vessels anatomy was compared with surgical findings in both groups.

**Results:**

The accuracy for detecting number of main renal arteries were not different in both groups which were 96.7% in CT angiography group and 90% in traditional angiography group (P = 0.15). The accuracy for detection of main renal veins were 100% in group 1 and 96.7% in group 2 (P = 0.31).

**Conclusions:**

MDCT has the same accuracy as traditional angiography to detect renal abnormalities in living kidney donors.

## 1. Background

Kidney transplantation is the treatment of choice for end-stage renal disease which can be donated from living or deceased donor. During recent years, interests to kidney transplantation from living donors have been obviously increased. Anatomical evaluation of the donor kidneys is essential to select the kidney to be used and to choose the appropriate surgical approach (1).

It has been shown that transplantation survival for kidneys from living donors, is higher than cadaveric sources (80% vs. 67% at 5 years) (2).

Traditional preoperative assessment included selective renal angiography. Development of multidetector helical CT (MDCT) angiography leads to perform this modality for living donors to decrease use of standard angiographic evaluation and its morbidities. According to previous studies, the accuracy of MDCT angiography for arterial anatomy was 93-97% (1-10).

## 2. Objectives

The aim of this study was to compare the accuracy of MDCT angiography and traditional angiography for evaluation of renal arteries in potential kidney donors. 

## 3. Patients and Methods

A total number of 60 consecutive kidney donors who underwent open surgical approach for transplantation were examined at our institute between Jan 2008 and Oct 2009. The donors were divided into two groups randomly. Thirty patients in group 1 underwent CT angiography which is our gold standard method and other thirty patients in group 2 underwent traditional angiographic examination (TAE) for preoperative evaluation. Kidneys were studied after the nephrectomy. All the subjects included in this study gave their informed consent. 

The mean age in group 1 was 39 years (19-55) and in group 2 was 38 years (20-53). 22 and 24 men were in group 1 and 2 respectively. All patients underwent open nephrectomy in our institute transportation program manner.

Patients in group 1 were examined by Phillip Brilliance 64-slice MDCT with 0.6 mm collimation 120 kVp 0.75 mm slice thickness and rotation speed of 33 gantroy. Sequence was performed after a delay of 20 arterials following the commencement of an infusion of 120-150 mL nonionic contrast medium (4 mL/s) via an antecubital vein. The time of maximum aortic enhancement in the era of read arteries was detected and scanning continued for 5 seconds after that time to view renal cortical perfusion. Patients in group 2 underwent standard selective renal angiography with femoral access. Renal vessels anatomy was compared with surgical findings in both groups. 

## 4. Results

Thirty patients in each group underwent open donor nephrectomy. As mentioned in Table 1, sensitivity, specificity and accuracy for detecting number of main renal arteries were 96.7% in group 1 and 90%, 100% and 90% in group 2, respectively (P = 0.15). Sensitivity, specificity and accuracy of two modalities for detecting early arterial branch was 100% in both groups (P = 0.21). Sensitivity and accuracy for detecting accessory artery were 50% and 93.3% in CT angiography group and 28.6% and 83.3% in selective angiography group respectively while specificity was 100% in both groups (P = 0.23).

Sensitivity and specificity and accuracy for determination of main renal veins were 100% in group 1 and 96.7% in group 2 (P = 0.31). 76.7% of gonadal veins were detected in group 1 compared with 22.3% in group 2 (P < 0.001). CT angiography and conventional angiography revealed 30% and 33.3% of lumbar veins respectively (P = 1). 40% of suprarenal veins were detected by CT angiography and 6.7% were detected by selective angiography (P = 0.005).

**Table 1.** Report of Sensitivity, Specificity and Accuracy and Comparison of Two Methods

	Sensitivity, %		Specificity, %		Accuracy, %		P value
	MDCT^a^	TAE^a^	MDCT	TAE	MDCT	TAE	
**Detecting Number Of Main Renal Arteries**	96.7	90	96.7	100	96.7	90	0.15
**Detecting Early Arterial Branch**	100	100	100	100	100	100	0.21
**Detecting Accessory Artery**	50	28.6	100	100	93.3	83.3	0.23
**Determination Of Main Renal Veins**	100	96.7	100	96.7	100	96.7	0.31

^a^ Abbreviation: MDCT, multidetector computed tomography; TAE, traditional angiographic examination

## 5. Discussion

Detection of the anatomy of renal arteries and confirmation of the absence of any parenchymal critical disease or tumors is essential for preoperative evaluation of potential renal donors. In a healthy donor the left kidney is usually harvested because of its longer pedicle. Digital subtraction angiography (DSA) has been used to recognize the number and length of rend arteries and assessment of unsuspected renal artery diseases such as atherosclerosis, aneurysm or fibromusculr dysplasia and renal parenchymal diseases such as cyst, scar or tumor.

Although DSA is an accurate technique for this proposes, it is invasive, expensive and accompanies with more major complications such as arterial perforation thrombosis and hematoma.

Development of CT technology currently allows faster scanning and MDCT scanners have provided more detailed data sets them single detector spiral CT. Three-dimentional CT angiography has been reported to be as accurate as renal angiography for arterial anatomy (7, 9).

CT scan with IV contrast also provide assessment of pyelocalyx system and ureteral anatomy so CT scan can be used as one step evaluation of potential kidney donors including evaluation of renal arteries veins and artery.

Anatomic information of renal artery is critical for donor nephrectomy. MDCT can well identify the number location presence of renal artery branching and renal arterial lesions such as atherosclerosis, aneurysm, fibromuscular dysphasia and ateriovenous malformation. Multiple renal arteries are the most important frequent finding in potential read donors and have been reported in 24-27% of renal arteriograms (11, 12).

Near half of the multiple rend arteries enter the polar region (13). The sensitivity of conventional angiography in visualizing total renal arteries in autopsy is 90% (12). 

Previous studies have been shown that CT angiography is a non-invasive technique with appropriate sensitivity and specificity to distinguish significant renal artery stenosis (14-18). Beregi et al. (15) showed that CT angiography had a sensitivity of 100% and 98% specificity for main renal artery stenosis. Limitation of CT angiography is in the diagnosis of fibromuscular hyperplasia and use of contrast media in it (19). 

Another new imaging modality which has been used for evaluation of kidney donors with reported sensitivity and specificity for main renal stenosis of 93-100% and 92-98% respectively is magnetic resonance angiography (20-24). Low ability to determine calcifications, high cost and limited access to MRI centers are limitations of MR angiography.

Zhang J et al. in their paper showed that multidetector-row computed tomography is helpful in accurately evaluating the renal anatomy of potential donors, thus facilitating planning of surgery (25).

Hänninen EL and colleagues in a study on 51 living kidney donors concluded that MDCT demonstrated superior accuracy compared with non-selective DSA for the preoperative assessment of renal anatomy in living kidney donors, and for the distinction of supernumerary arteries versus early branching patterns, 16-channel CTA data were better than those of the four-channel system (26).

Kawamoto S et al. in their paper mentioned that multi-detector row CT scanners offer shorter image acquisition time, narrower collimation, better spatial resolution, and less tube heating than single-detector row CT scanners. Multi-row scanners also provide more complete anatomic coverage, increased contrast enhancement of the arteries, and greater longitudinal spatial resolution, all of which are important both for accurate imaging of the renal vasculature and for three-dimensional post processing of image data. They also recommended making the most effective use of this method; radiologists must be familiar with its technical aspects, advantages, and potential pitfalls. They also must be able to identify variations in vascular, renal and extra renal anatomy that are important for laparoscopic donor nephrectomy (27).

In conclusion, CT angiography is an accurate, safe and non-invasive imaging modality for detection of renal artery and vein abnormalities in potential living kidney donors. MDCT has the same accuracy as traditional angiography for detecting renal abnormalities in living kidney donors.
